# From Human Papillomavirus (HPV) Detection to Cervical Cancer Prevention in Clinical Practice

**DOI:** 10.3390/cancers6042072

**Published:** 2014-10-02

**Authors:** Sin Hang Lee, Jessica S. Vigliotti, Veronica S. Vigliotti, William Jones

**Affiliations:** Department of Pathology, Milford Hospital, 300 Seaside Ave., Milford, CT 06460, USA; E-Mails: jessvigs@yahoo.com (J.S.V.); ronnie313vig@yahoo.com (V.S.V.); will.jones@milfordhospital.org (W.J.)

**Keywords:** reliable HPV genotyping, DNA sequencing, persistent HPV infections, cervical screen, cervical cancer, pap smears, cervical cytology, cervical cancer prevention

## Abstract

The newly gained knowledge of the viral etiology in cervical carcinogenesis has prompted industrial interests in developing virology-based tools for cervical cancer prevention. Due to the long incubation period from viral infection to developing an invasive cancer, a process whose outcome is influenced by numerous life-style and genetic factors, the true efficacy of the genotype-specific human papillomavirus (HPV) vaccines in cervical cancer prevention cannot be determined for another 30 years. Most HPV DNA test kits designed to replace the traditional Papanicolaou (Pap) smears for precancer detection lack the analytical sensitivity and specificity to comprehensively detect all potentially carcinogenic HPVs and to perform reliable genotyping. The authors implemented the classic nested PCR and Sanger DNA-sequencing technology for routine HPV testing. The results showed a true negative HPV PCR invariably indicates the absence of precancerous cells in the cytology samples. However, 80.5% of single positive HPV-16 tests and 97.3% of single positive HPV-18 tests were associated with a negative or a largely self-reversible Pap cytology. Routine sensitive and reliable HPV type-specific or perhaps even variant-specific methods are needed to address the issues of persistence of HPV infection if a virology-based primary cervical screen is used to replace the Pap cytology screening paradigm.

## 1. Introduction

Persistent infection by certain DNA viruses, *i.e.*, the high-risk (carcinogenic) types of the genus alphapapillomavirus of the papillomaviridae family, commonly known as human papillomavirus (HPV), is a necessary factor in the pathologic process which may lead to cervical cancer development [1,2,3,4]. Cervical cancer is a major fatal malignancy among women, causing about 275,000 deaths annually worldwide, mostly in developing countries [5].

Cervical cancer is also a highly preventable disease if detected at its precancerous stages and treated by ablative procedures. Since there are no treatments to cure the persistent HPV infections, prevention of cervical cancer is accomplished by physical removal of the cancer-susceptible transformation zone of the uterine cervical epithelium when a biopsy shows the presence of high-grade cervical intraepithelial neoplasia (CIN3), representing pathologic changes with a high frequency of progressing to invasive cancer. In the United States, the widespread use of Papanicolaou (Pap) smear screening for detection followed by treatment of these precancerous lesions reduced the incidence of cervical cancer from 44 in 100,000 women in 1947 to 8.8 in 1970 [6]. About 79 million American women are infected with HPV with about 14 million becoming newly infected each year [7]. In 2010, there were 11,818 American women diagnosed with cervical cancer, and 3939 cervical cancer deaths [8]. The age-standardized mortality rate is 1.7 per 100,000 [5]. Cervical cancer is primarily a disease among unscreened or rarely screened women [9].

The discovery of DNA viruses as a major factor in causing most of the cervical squamous cancers has led to new hopes to use a virology test for cervical screening of precancerous lesions and to use an HPV vaccine for cancer prevention [10]. A commercial HPV DNA detection kit was first introduced to detect high-risk HPV genotypes, *i.e.*, HPV-16, 18, 31, 33, 35, 39, 45, 51, 52, 56, 58, 59 and 68 for cervical screening [11,12]. Since 2006, two protein-based vaccines containing virus-like particles (VLPs) as the active ingredient have been available for prevention of the infection by the two most common high-risk HPV genotypes, *i.e.*, HPV-16 and HPV-18, with the aim to prevent cervical cancer in the subjects.

The VLPs in the vaccines are irregularly shaped 30–50 nm structures composed of self-assembled HPV major capsid L1 protein pentamers manufactured by a DNA recombinant technology [13,14]. Genotype-specific VLPs for HPV-16, -18, -11 and -6 are used as the active ingredient of a quadrivalent HPV vaccine Gardasil^®^ (Merck) and those for HPV-16 and HPV-18 are used as the active ingredient of a bivalent HPV vaccine Cervarix^®^ (GlaxoSmithKline). After being injected intramuscularly with an aluminum salt adjuvant, HPV VLPs are exceptionally effective in eliciting production of neutralizing antibodies against HPV-16 [15,16,17]. In clinical trials, these vaccines have been shown to reduce the incidence of cervical intraepithelial neoplasia grade 2 (CIN2) and grade 3 (CIN3) in vaccinated women [18]. Mass vaccination of young females has been proposed as a means for cervical cancer prevention [10]. However, since the CIN2/CIN3 lesions were used as the surrogate endpoint in the clinical trials [18,19] and are self-reversible lesions to varying degrees [20,21,22], and since the median age at diagnosis for HPV-associated cervical cancer is 48 years [23], the true efficacy of HPV vaccination against cervical cancer cannot be determined with any certainty in a vaccinated population until another three decades have past. In addition, vaccination of young women already infected with a vaccine-relevant HPV genotype may accelerate the development or progression of a precancerous lesion [19]. Sporadic cases of invasive cervical cancer after HPV vaccination have been reported [24], raising a concern about the policy of dependence on vaccination alone as a means for cervical cancer prevention. Recently, other less common HPV genotypes, for example, HPV-26, 30, 61, 67, 68, 69, 73 and 82 which were not considered to be “carcinogenic” have been found in invasive cervical cancer tissues [25], suggesting a complex and still poorly understood relationship between infection by various specific HPV genotypes and cervical carcinogenesis. Both American Cancer Society [26] and the National Cancer Institute [27] advise that all women should continue to follow the guidelines of cervical screening regardless of their HPV vaccination status. Post-licensure monitoring evaluations showed that the quadrivalent genotype-specific HPV vaccine is effective in preventing the vaccine-type HPV infection among females aged 14–19 years, but not among females aged 20–49 years [28].

At the present time, detection of persistent high-risk HPV infection and cytologic monitoring of the precancerous changes, if present, are the two screening tests which the practitioners rely on for patient management in cervical cancer prevention. The U.S. FDA has recently approved using a human papillomavirus test for primary cervical cancer screening for women of 25 or older [29].

In a normal life cycle, when a high-risk HPV infects the basal epithelial cells of the cervical epithelium, the early HPV genes, *i.e.*, the E1, E2, E4, E5, E6 and E7 genes, are expressed as viral DNA replicates from the episomal DNA. The late genes, L1 and L2, are expressed in the cells of the upper epithelial layers to generate the L1 and L2 capsid proteins to encapsulate the viral genomes to form virions. The proteins E6 and E7 are known to inhibit the tumor suppressor genes p53 and pRb, and the protein E5 appears to have a weaker oncogenic property which results in increased activity of epidermal growth factor receptor (EGFR) [30]. However, the infected cells usually die upon viral particle formation.

The proteins E2 and E1 play a leading role in control of transcription and replication, and the absence or loss of activity of these proteins leads to deregulation of E6 and E7 oncoproteins [31]. If the viral genome is integrated, its DNA is damaged and E7 and E6 gene expression is no longer controlled by virus [32]. Under this condition, persistent infection with some of the high-risk HPV types can induce progression of the epithelial dysplastic changes to CIN3 in 5–10 years [33]. Development of cellular dysplastic changes or a truly cancerous pathology is influenced by numerous factors, such as smoking habit, immune suppression, oral contraceptive use, and hormone replacement therapy [34] as well as host genetic makeups [35,36]. Progression of the initial cytopathologic process to micro invasive and invasive cancer usually takes decades [33]. Today, we know of more than 200 HPV genotypes [37] of which about 40 are routinely isolated from the female lower genital tract [38].

HPV has a genome of about 8000 base pairs (bp) consisting of a covalently closed circular double-stranded DNA molecule. By definition, a genotype of HPV differs in the L1 gene DNA sequence, which is about 1600 bp in size and highly conserved, by at least 10% from every other known HPV type; HPV subtypes are those having DNA sequence similarities between 90% and 98% with a prototype in its L1 gene; and variants of an HPV genotype are those having a DNA sequence identity of over 98% of a prototype [39]. Since sequence dissimilarities are unevenly distributed with scattered short sequence homologies between sequence variants of different HPV genotypes positioned in various segments of the L1 gene [40], clinical laboratories usually determine a highly conserved sequence with hypervariable regions of a short target DNA sequence in the L1 open reading frame (ORF) of the viral genome for HPV genotyping after the short target DNA segment is amplified by polymerase chain reaction (PCR), using a pair of degenerate or general PCR primers for the cyclic primer-directed DNA synthesis [38,41,42,43].

The presence of HPV episomal DNA in the epithelial cells is often associated with koilocytotic atypical changes as the manifestation of HPV-induced cytopathology in cervical dysplasia [44], a reversible early precancerous pathology which can be recognized on Pap smears and in histopathologic sections as low-grade cervical intraepithelial neoplasia (CIN1), the precursor stage in the development of a CIN2 and a CIN3 lesion. This link between a virology finding and cytopathic changes in cervical carcinogenesis has led to the proposed guidelines of using HPV DNA testing to replace the traditional Pap smear cytology as the primary screening for referral to colposcopy of HPV-16/HPV-18-positive women with negative cytology for cervical cancer prevention [29,45].

As much as 99.7% of the cervical cancers studied have been found HPV-positive in some reports [46]. However, most HPV-infected epithelial cells do not show precancerous changes, and the viral infection can be reversed, suppressed or eliminated through innate immunity of the host and other life-style factors with no residual adverse health consequences. It is the persistent infection of a high-risk HPV, not the mere presence of the HPV virus itself, that is the pivotal promoter in causing cervical precancerous lesions and cancer [1,2,3,4]. Most HPV infections, even caused by high-risk genotypes, are transient with normal Pap cytology in sexually active young women [47,48,49,50]. In 93% of initially infected women, the same viral type is not detected upon re-examination four menstrual cycles later [51]. The median duration of positivity detectable by PCR for a specific HPV type in these young women is 168 days [52]. Multiple high-risk HPV infections do not constitute a higher risk for the development of cervical neoplasia (CIN1-3) when compared with single persistent high-risk HPV infection [53]. For the development and maintenance of a CIN3 lesion, the risk is greatest in women positive for the same genotype of HPV on repeated testing [1,2,3,4].

Viral load is not a useful parameter to predict CIN3 or cancerous lesions [54] because HPV DNA integration itself is followed by a decrease in viral load in the host cell. As the pathology of persistent HPV infection advances from a low-grade CIN1 lesion which often contains numerous large koilocytes, to a CIN2/CIN3 lesion or a true malignancy, the size of the abnormal cells, their cytoplasm-to-nucleus ratio, and the viral load per abnormal cell all tend to decrease simultaneously [55,56]. A high-grade CIN lesion is often associated with a viral DNA load lower than that observed in less severely affected cells [55] although during the course of persistent infection there may be an increase in average viral load per cell in a collected Pap cytology sample at certain stages while the infected cells are progressing from a low grade to a high grade CIN3 lesion [57,58]. Based on studies of keratinocyte cultures infected by HPV-16, it is estimated that the small cells contain approximately 100 episomal copies of HPV DNA per cell, whereas the large cells contain approximately 3,500 copies per cell [59]. The well-established SiHa cervical cancer cell line contains only one to two copies of HPV-16 DNA per cell [60]. Therefore, the HPV DNA test used to replace the traditional Pap cytology screening for precancerous cells and cancer cells in the cervicovaginal samples must be highly sensitive, capable of detecting not only the HPV DNA with high copy number in the koilocytes of the low grade CIN lesions, but also the HPV DNA in the cervical cancers which may have as low as one single copy of HPV DNA per cancer cell, and to generate a reliable genotyping result of the detected HPV. Cancer cells in Pap cytology samples are usually low in number, and require specially trained cytotechnologists for their detection.

Most commercially available HPV test kits have been shown to be suboptimal in analytical sensitivity and typing specificity. It has been reported that in a comparative study one of the approved HPV DNA test kits on the market was found to generate two to four times more positive results than the other [61]. In a 2010 WHO report of HPV Global Proficiency Study on 98 laboratories worldwide with a total of 132 data sets submitted for analysis, only 26 data sets proficiently detected all 16 coded HPV types [62]. The major oncogenic HPV types, 16 and 18, were proficiently detected in 95.0% (114/120) and 87.0% (94/108) of the data sets, respectively. In 118 test sets, 39% (46/118) were found to be not proficient in detecting the specific HPV types. At least one false-positive result was reported in 46% (61/132) test sets. Among the 17 data sets generated by the most commonly used Linear Array (Roche) kit, only 47% (8/17) were 100% proficient of detecting the HPV types tested for, whereas none of the 12 data sets using the InnoLiPA (Innogenetics) kit were 100% proficient [62]. The study was designed to evaluate and to improve the performance of HPV typing tests used in HPV vaccinology and HPV surveillance. In the opinions of these authors, high analytical sensitivity is needed in vaccinology, but not in cervical cancer screening programs [62]. The need to create such a double standard in HPV testing—one for the vaccine manufacturing industry and one for the cervical cancer screening industry—is questionable in practicing science-based personalized medicine.

Using a nested PCR amplification for detection followed by automated DNA sequencing for genotyping as the standard for validation, the high-risk HC2 test (Digene/Qiagen) detected only 57.6% (388/674) of the high-risk HPV isolates in clinical specimens, mislabeled 46.8% (88/188) of the low-risk HPV isolates as high-risk genotypes, and misclassified 27.4% (180/657) of the negative samples as being infected by high-risk HPV [63]. Based on the current understanding of the HPV-initiated cervical carcinogenesis, a total shift from the traditional cytologic screening paradigm to HPV screening by available commercial test kits as a means for cervical pathology detection is probably premature, and this issue can be adequately studied only when highly sensitive HPV genotype-specific or perhaps even variant-specific methods are available to the clinical laboratories which perform routine HPV tests for patient management. In 2007, we began to use a nested PCR detection followed by Sanger DNA sequencing, the recommended “gold standard” for evaluation of all HPV nucleic acid test kits [64], for routine HPV genotyping to assist the gynecologists in private practice to manage the pathology detected in cervical screening in a representative rural and suburban population of the USA [65,66,67]. In this paper, we summarized and analyzed the data compiled from 2007–2010 to explore the potential of using a nested PCR/DNA sequencing-based HPV DNA assay as an analytically sensitive and specific HPV screening in the management of the cervical pathology of patients under the care of gynecology specialists in private practice.

## 2. Results and Discussion

Recently, many studies worldwide have reported failures of detecting HPV DNA in some histologically confirmed cases of invasive cervical cancers, including squamous cell carcinoma. For example, 8% of patients with cervical squamous cell carcinoma were found to be HPV-negative within 30 months preceding the histological diagnosis of a cervical squamous cell carcinoma [68]; and 12.6% of histologically confirmed cases of cervical carcinoma were HPV-negative [69]. Based on the analysis of large-scale data from 17 European countries, laboratories using the PCR-based SPF10-LiPA25 test kit did not find HPV DNA in 8.2% of histologically proven invasive cervical cancers [70]; and no HPV DNA was detected in 13% (1234/9486) of confirmed cases of squamous cell carcinoma [71]. The finding of so many HPV-negative cervical cancer cases contradicts the fundamental teaching that HPV is a necessary factor in cervical carcinogenesis, and raises the possibility that the commercial test kits might have failed to detect some of the less known carcinogenic HPV types in the clinical specimens at the precancerous stage. A testing method with high analytical sensitivity and specificity for patient management may help monitor the patients with persistent infection by a less common “carcinogenic” HPV genotype or variant which is not included in the fixed number of HPVs targeted for detection in the commercial HPV test kits.

We have modified the protocol of primary PCR amplification with a pair of MY09 and MY11 degenerate primers followed by heminested PCR with a pair of consensus GP6 and MY11 primers at low stringency PCR condition for HPV detection and performed a routine Sanger DNA sequencing on all nested PCR amplicons for HPV genotyping. This nested PCR protocol is known to be able to amplify a single copy of the highly conserved HPV L1 gene DNA fragment with hypervariable base regions [1,65] and the PCR amplicon can be used for routine direct automated DNA sequencing for genotype validation. Our experience in implementation of this technology at a point-of-care community hospital laboratory is presented as follows.

### 2.1. Nested PCR and Direct DNA Sequencing Can Detect Minor HPV Variants

**Figure 1:**
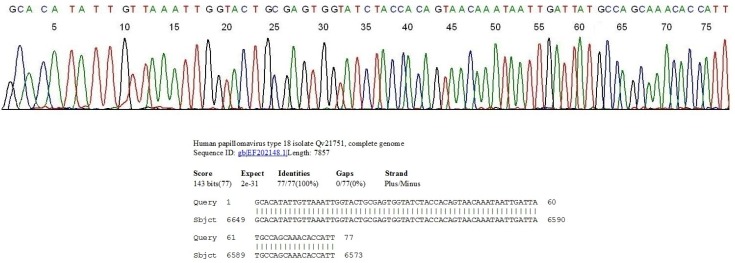
Segment of the computer-generated base-calling electropherogram showing a DNA sequence of the GP6/MY11 PCR amplicon of a European or Asian-American subtype of HPV-18 in a cervicovaginal cell suspension, and the BLAST validation report from the GenBank. The sequence CCATT on the far right represents the 3' end of the MY11 primer. Note the sequence GCAAACA immediately downstream of the MY11 primer, characteristic of the European or Asian-American subtype of HPV-18.

**Figure 2:**
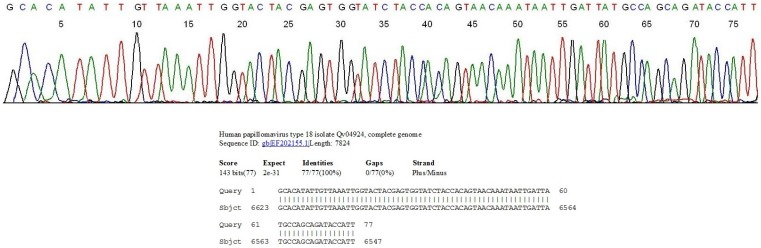
Segment of the computer-generated base-calling electropherogram showing a DNA sequence of the GP6/MY11 PCR amplicon of an African subtype of HPV-18 in a cervicovaginal cell suspension, and the BLAST validation report from the GenBank. The sequence CCATT on the far right represents the 3' end of the MY11 primer. Note the sequence GCAGATA immediately downstream of the MY11 primer, characteristic of the African subtype of HPV-18.

Due to the diversity of the L1 gene DNA sequences between the individual HPV genotypes, the use of type-specific primer PCR to amplify all possible clinically relevant HPV genotypes individually on each clinical sample is impractical and uneconomical. For PCR detection of HPV DNA in clinical specimens, specificity is sacrificed for high sensitivity and efficiency to detect all possible clinically relevant genotypes, including the less frequently encountered variants or mutants, with one set of consensus general primers. The target DNA can be validated by DNA sequencing of the inter-primer segment of the PCR amplicon. For example, there are three subtypes of HPV-18, namely the European, the Asian-American and the African subtypes [72]. All of the HPV-18 isolates from European women were found to be those of the European or Asian-American variants, both sharing an identical DNA sequence in this target segment [73]. In the United States, 91% of the HPV-18 isolates from white women were reported to be of the European/Asian-American variants, and 64% of the HPV-18 isolates from African American women belong to the African variants [74]. Using the comprehensive L1 gene amplification by the degenerate consensus MY09/MY11 and GP6/MY11 heminested PCR primers, followed by automated direct DNA sequencing, both the European/Asian-American subtype (Figure 1) and the African subtype (Figure 2) of the HPV-18 were detected.

Since there is a two-nucleotide-base difference in DNA sequence immediately downstream of the MY11 primer between the European/Asian-American subtype and the African subtype of HPV-18, a commercial HPV assay kit designed to target one subtype for hybridization may fail to detect the other [75], and this sequence difference might have contributed to a lower proficiency rate in testing simulated clinical samples containing HPV-18 (87.0%) than the rate when HPV-16 was the test target (95.0%) [62]. In the author’s laboratory serving a female population under the care of private gynecologists in southern Connecticut, about 80% of the HPV-18 isolates from the clinical samples are of the European/Asian American subtype with about 20% being those of the African subtype [75].

As previously reported, our routine work with DNA sequencing unveiled a novel HPV-39 variant with an A-to-C mutation, thus changing the 14th amino acid from leucine (TTA) to valine (GTA) in the L1 major capsid protein; and this mutant was repeatedly isolated from a patient with persistent HPV infection and a history of precancerous cytopathology [66]. Another novel HPV-87 isolate with seven nucleotide mismatches in the inter-primer region without altering the amino acid sequence of the L1 capsid protein in this region was also described [67]. These infrequent HPV or novel variants would not have been detected by using any of the commercial test kits which have been designed to test for prevalent or standard sequences of the HPV prototypes listed in the GenBank only. Among the high-risk genotypes, failure to detect HPV-18 is of a special concern since HPV-18 may be more oncopotent than HPV-16 in uterine cervical carcinogenesis in certain populations [76].

### 2.2. HPV Genotyping and Cytopathology in Samples Collected in Private Practice

From 2007–2010, the department of pathology of Milford Hospital in Milford, Connecticut, USA received and processed 8461 alcohol-preserved liquid-based cervicovaginal Pap cytology specimens (ThinPrep System; Cytyc, Marlborough, MA, USA; or Surepath, BD Diagnostics, Franklin Lakes, NJ, USA) collected by board-certified gynecologists in private practice with request for HPV testing according to the American College of Obstetricians and Gynecologists guidelines [77].

The HPV tests were ordered as part of regular medical office women’s health care for patients aged 30 years and older as adjunctive screening to routine Pap cytology, and for patients below 30 years who had a cytologic diagnosis of atypical squamous cells of undetermined significance (ASCUS) or more severe cytological changes in most of the cases. This study was a retrospective analysis of laboratory data and the data analysis presented here did not influence the decision of patient management. Publication of these laboratory data with blinded patient identities was approved by the Milford Hospital Institutional Review Board as an ongoing research project [66].

The Pap cytology screening was performed by an independent commercial laboratory (Quest Diagnostics Laboratory, Wallingford, CT, USA), and all abnormal cytology slides classified as ASCUS, low grade squamous intraepithelial lesion (LSIL) or high grade squamous intraepithelial lesion (HSIL) were reviewed by a board-certified pathologist for final classification. Detection of HPV DNA by GP6/MY11 heminested PCR followed by direct automated DNA sequencing for genotyping was performed according to the method previously published [65,66,67], and the results of the concomitant HPV test and Pap cytology screen on 8461 split samples are summarized in Table 1.

**Table 1:** Results of HPV test and Pap cytology on 8461 split cervicovaginal cell samples ordered by private gynecologists in Milford, CT, USA.

HPV Genotype	No. PAP-Negative	No. PAP-ASCUS	No. PAP-LSIL	No. PAP-HSIL(%)	[(PAP− cases)/(HPV+ cases)] = (%)
**6**	17	7	6	0	17/30 (56.7)
**11**	1	1	1	0	1/3 (33.3)
**16**	55	16	20	22(19.5)	55/113 (48.7)
**18**	19	6	11	1(2.7)	19/37 (51.4)
**26**	0	0	3	0	0/3 (0)
**31**	15	11	7	5(13.2)	15/38 (39.5)
**32**	5	0	0	0	5/5 (100)
**33**	10	4	2	3(15.8)	10/19 (52.6)
**35**	13	2	6	2(8.7)	13/23 (56.5)
**39**	17	4	8	0	17/29 (58.6)
**40**	3	2	0	0	3/5 (60.0)
**44**	6	3	0	0	6/9 (33.3)
**45**	22	5	3	1(3.2)	22/31 (71.0)
**51**	3	0	3	0	3/6 (50.0)
**52**	31	7	12	4(7.4)	31/54 (57.4)
**53**	5	1	4	0	5/10 (50.0)
**54**	40	2	2	0	40/44 (90.9)
**55**	4	2	0	0	4/6 (66.7)
**56**	9	4	5	0	9/18 (50.0)
**58**	15	4	4	0	15/23 (65.2)
**59**	18	3	8	1 (3.3)	18/30 (60.0)
**61**	19	4	1	0	19/24 (79.2)
**62**	19	0	2	0	19/21 (90.5)
**66**	2	3	15	0	2/20 (10.0)
**67**	5	3	1	0	5/9 (55.6)
**68**	2	0	1	0	2/3 (66.7)
**69**	2	1	1	0	2/4 (50.0)
**70**	11	0	2	0	11/13 (84.6)
**71**	2	0	0	0	2/2 (100)
**72**	17	0	0	0	17/17 (100)
**73**	15	6	6	0	15/27 (55.6)
**74**	1	1	0	0	1/2 (50.0)
**81**	16	3	7	0	16/26 (61.5)
**83**	2	1	0	0	2/3 (66.7)
**84**	12	0	0	0	12/12 (100)
**86**	1	0	1	0	1/2 (50.0)
**87**	1	1	1	0	1/3 (33.3)
**89**	1	0	1	0	1/2 (50.0)
**90**	1	0	0	0	1/1 (100)
**91**	1	0	0	0	1/1 (100)
Mixed	18	17	5	2 (4.8)	18/42 (42.9)
HPV+ cases Subtotal	456	124	149	41	456/770 (59.2)
HPV− cases (PAP− %)	7545	146	0	0	7545/7691 (98.1%) (<LSIL 100%)
Total No.	8001	270	149	41	8461

Note: The 40 HPV genotypes found in a Southern Connecticut female population in boldfaced numbers are listed in the left column and the 13 high-risk HPV genotypes are underlined. The Pap cytology results classified as Negative, ASCUS, LSIL and HSIL of the split samples of each HPV genotype-positive cases are tabulated on the right to each HPV genotype. The total number of samples positive for each HPV genotype is shown as denominator in the far-right column with the number of Pap negative cases listed as numerator followed by a % in (). The overall % of specimens positive for HPV is 9.1% (770/8461); and 59.2% (456/770) of the HPV-positive samples were negative for Pap cytology. Of the 37 HPV-18 isolates, 30 belonged to the European/Asian American subtypes (Figure 1) and 7 to the African subtype (Figure 2).

The data presented in Table 1 show that a negative HPV nested PCR had a predictive value of 98.1% (7545/7691) for a negative Pap cytology result. About 2% of the HPV-negative cases were interpreted as ASCUS and needed clinical follow-ups. Of the 770 HPV-positive patients, 626 had at least one follow-up HPV test 6 months to 3 years after the initial test at entrance, including 372 patients whose follow-up HPV test was negative and 25 patients whose follow-up HPV tests showed an HPV genotype which was different from the genotype found at the entrance test. These latter two groups of patients (372 + 25 = 397) did not have a Pap cytology class >ASCUS on the follow-up tests, and were considered having a transient HPV infection at the entrance, and therefore to be “free of cervical cancer risk”. The follow-up samples from 229 of the 626 patients were positive for an HPV genotype identical to the one detected at entrance; these patients were considered having a persistent HPV infection to be analyzed further below. The remaining 144 HPV-positive patients did not have follow-up tests primarily because the initial HPV detected was a low-risk genotype and the Pap cytology was below the LSIL class.

There were no LSIL or HSIL cytology results associated with a negative HPV PCR. Our findings support the conclusion made by others that HPV infection is a necessary factor for initiating the pathologic process which may lead to cervical cancer [1,2,3,4]. A negative HPV result practically assures an absence of precancerous or cancer cells in the cervicovaginal sample. No invasive cancer has been recorded in this patient population under study [66]. As repeatedly pointed out by Stoler, a leader in the HPV testing industry, an “extremely sensitive” PCR-based method can be used as the first step in a cervical cancer screening program [78,79]. The results summarized in Table 1 show that the heminested PCR technology fulfills this “extremely sensitive” requirement.

On the other hand, a positive high-risk HPV DNA test is not consistently associated with a precancerous or cancerous Pap cytology. A negative Pap cytology was found in a low 39.5% of HPV 31-positive samples to a high 71.0% of HPV 45-postitive samples. When HPV-16 was detected on single tests at the time of entrance in this study, only 19.5% (22/113) of the split samples were found to have a HSIL cytology, an indication for immediate colposcopic biopsy to rule out cancer or an advanced precancerous CIN3 lesion. The other HPV genotypes associated with a HSIL cytology were HPV-33 with 15.8% (3/19) HSIL, HPV-31 with 13.2% (5/38) HSIL, HPV-35 with 8.7% (2/23) HSIL, HPV-52 with 7.4% (4/54) HSIL, HPV-59 with 3.3% (1/30) HSIL, HPV-45 with 3.2% HSIL, and HPV-18 with 2.7% (1/37) HISL. An HSIL cytology was found in 4.8% (2/42) mixed HPV infections. This latter finding was not a surprise because genotype-specific PCR amplification followed by automated DNA sequencing for validation has confirmed that 82% of the cervicovaginal cell samples infected by multiple HPV genotypes found by nested PCR in fact contained an HPV-16 in addition to various other companion genotypes [63]. In the current series, most patients with an ASCUS or a LSIL cytology were clinically observed without an immediate colposcopic biopsy. Among the high-risk genotypes, HPV-18 had the lowest percentage of HSIL cytology cases in this female population.

The findings presented above indicate that a reliable negative HPV test can eliminate the need for a Pap cytology test. However, a single positive HPV test alone is not a good indication for colposcopic biopsy because 80.5% (91/113) of the HPV-16 positive samples and 97.3% (36/37) of the HPV-18 positive samples were associated with a negative Pap cytology or a largely self-reversible Pap cytology in the category of ASCUS or LSIL. Using an FDA-approved industry-standard test kit for triage of patients, more than 95% of referrals to colposcopic biopsies for diagnostic workup based on a single positive high-risk HPV DNA test result have been found to be unnecessary and potentially excessive [80].

Recently, in an attempt to reduce the number of unnecessary and excessive 4-quadrant colposcopic biopsies the supporters of using commercial HPV DNA test kits for triage of patients to biopsy workup have introduced a pair of clinical sensitivity and clinical specificity targets as the “gold standard” for validation of novel HPV test kits [81,82]. One group recommended that any HPV DNA assay kit must have a clinical sensitivity and clinical specificity for high-grade CIN or worse (CIN2) of not less than 90% and 98% of those of HC2, respectively [81]. Another group proposed a clinical sensitivity and clinical specificity for CIN3 of 92% ± 3% and at least 85%, respectively [82]. However, since CIN 2 is not a true biologic entity but an equivocal diagnosis of precancer, in fact representing an admixture of HPV infection and precancerous changes and a range of observational variables [21], and since 19%–50% of the CIN2 and CIN3 lesions are self-reversible [20,21,83,84,85], it is extremely difficult if not impossible to use such a moving standard to evaluate HPV DNA assays which are based on DNA science obeying the laws of physics. Currently, all commercial test kits depend on selecting a cutoff point to separate the positive and negative results, by raising the positive/negative cutoff point in the signal reading scale to increase the “clinical specificity” at the expense of “clinical sensitivity” or by lowering the cutoff point to increase the “clinical sensitivity” at the expense of “clinical specificity”. When the HPV-positive rate generated by the Cervista test kit was found to be 2–4 times that of the HC2 test kit [61], it was difficult to decide if the Cervista test kit had failed to meet the acceptable “clinical specificity”, or the HC2 test kit failed to meet the “clinical sensitivity”, or both test kits had failed the expected clinical sensitivity and clinical specificity. Our findings suggest that it is impossible to rely on one HPV test kit for primary cervical cancer screening and for triage of patients to further cancer workup without causing excessive and unnecessary colposcopic biopsies or missing potential precancerous cases which need follow-ups for cancer prevention. None of the available HPV test kits can meet the “extremely sensitive” requirement for primary cervical screening [78,79]. Our data confirmed that a negative nested HPV DNA test practically assures no concomitant LSIL or HSIL Pap cytology in the samples, in line with the “extremely sensitive” HPV test recommendation by Stoler [78,79]. We found no published data to link a specific viral load of any HPV genotype to a LSIL or a HISL Pap cytology. Using a single high-risk HPV DNA test for triage of patients to cancer workup may be referring many patients with negative Pap cytology to colposcopic biopsies even when the HPV test results are analytically specific and validated by genotyping with direct Sanger sequencing.

The FDA-approved Roche Cobas 4800 HPV kit used for detection of ≥CIN 2 and ≥CIN 3 in an ASCUS population was reported to have a clinical sensitivity 90.0% and a clinical specificity 70.5% for ≥CIN 2 lesions, and a clinical sensitivity 93.5% and clinical specificity 69.3% for ≥CIN 3 lesions [86]. These performance characteristics are below the “extremely sensitive” requirement originally recommended by Stoler for HPV DNA cancer screening [78,79], and do not meet the criteria of “a clinical sensitivity and clinical specificity for CIN3 of 92% ± 3% and at least 85%, respectively” [82]. For primary cervical cancer screening, a “clinical sensitivity” of 90.0% is too low because screening with these test kits would misclassify as “negatives” 10% of the patients who may need a follow-up for cervical cancer prevention. Lowering the cutoff point to increase the “clinical sensitivity” will reduce the “clinical specificity” with the consequence of referring more patients to unnecessary and excessive colposcopic biopsies.

### 2.3. Correlation of HPV Genotyping, HSIL Cytology and Histologic Findings

According to the existent guidelines of practice, patients with a HSIL cytology (Table 1) were advised to undergo immediate colposcopic four-quadrant biopsies, and the results are presented in Table 2.

**Table 2:** HPV genotypes in 41 patients with HSIL cytology and their colposcopic biopsy results.

HPV Genotype	Four-Quadrant Biopsies					
	Negative	CIN1	CIN2	CIN3	No Biopsies *	Total
16	0	4	4	11	3	22
18	0	1	0	0	0	1
31	1	2	1	1	0	5
33	1	0	1	1	0	3
35	0	0	1	1	0	2
45	0	1	0	0	0	1
52	0	0	2	2	0	4
59	0	0	1	0	0	1
Mixed	1	1	0	0	0	2
Total	3	9	10	16	3	41

* Patients moved out of area or refused consent to biopsy.

The data in Table 2 showed that patients with a single positive HPV-16 and a concomitant HSIL cytology had a 50% (11/22) probability of harboring a CIN3 lesion, an 18.2% (4/22) probability of a CIN2 and an 18.2% (4/22) probability of a CIN1 lesion, confirmed by histopathology after colposcopic biopsy. The only other five histologically confirmed CIN3 lesions were observed in patients with a positive HPV-31, HPV-33, HPV-35 and HPV-52 along with a HSIL cytology. However, the numbers were too small for a meaningful analysis in this patient population.

Based on data generated by laboratories using various test kits, the overall HPV prevalence in women with normal cervical cytology worldwide was estimated to be 10.4% [87], compared to the 9.1% positivity in this patient population (Table 1). High-risk HPV screening tests have been shown repeatedly to have a higher sensitivity, but a lower specificity than the traditional Pap cytology test, in detecting histologically confirmed CIN2, and CIN3 lesions [88,89,90]. However, the clinical significance of histologically confirmed CIN2 lesions is debatable because its diagnosis is highly subjective and frequently self-reversible [20,21,83,84,85]. Of the women 30 years or older whose cervicovaginal cells were negative for Pap cytology test, but positive for HPV-16 and/or HPV-18, some were found to carry a CIN3 lesion or a true cancer in the cervix [45], suggestive of a higher sensitivity of the HPV testing than the traditional Pap cytology in cervical screening for cancer prevention. However, when invasive cervical cancer tissues were analyzed for the presence of HPV genotypes, some of the cancers were found to contain HPV genotypes which are not generally considered high-risk or carcinogenic [25], indicating that the current commercial HPV test kits targeting about 13 high risk HPV genotypes for detection may not be comprehensive enough to unveil all HPV genotypes capable of causing cervical cancers.

In a head-to-head comparative study performed on split clinical samples, using nested PCR for detection followed by direct DNA sequencing for HPV genotyping as the standard for comparison, an FDA-approved high-risk HPV test was found to correctly detect 57.6% of the high-risk HPV isolates in clinical specimens, mislabel 46.8% of the low-risk HPV isolates as high-risk genotypes, and classify 27.4% of the “true-negative” samples as being infected by high-risk HPV [63]. Therefore, a highly sensitive and HPV genotype-specific test, similar to those used in medical research [1], is needed at the point of care for furthering our knowledge while trying to translate the science of viral carcinogenesis into practice for cancer prevention.

### 2.4. HPV Genotype Persistence and Cytohistopathologic Dynamics

In this series, there were 229 patients who had at least one additional positive HPV test showing the same genotype in the second sample taken between 6 months and 3 years after the first sample was tested, thus considered having a persistent HPV infection, and at least one follow-up cytology test associated with the second HPV assay. Comparing the subsequent cytology with the first, these patients were placed into three classes, namely no change (NC), regress to lower class (REG) or progress to higher class (PRO) in Pap cytology. These follow-up results in cytology were tabulated (Table 3) against the individual HPV genotypes found in the persistent infections. The results of the 4-quandrant colposcopic biopsies, if performed, after the follow-up HPV/cytology tests, were also listed.

As shown in Table 3, the initial Pap cytology samples in this group of patients at entrance were classified as negative (NEG), ASCUS (ASC) and LSIL, all positive for various HPV genotypes validated by L1 gene DNA sequencing. Patients with a HSIL cytology result were excluded for the follow-up analysis because they were referred to immediate colposcopic biopsy.

**Table 3:** Genotype-specific HPV persistent infections correlated with follow-up cytology and biopsy.

HPV Type	Initial Cytology Classification			Follow-up Cytology			Biopsy Result		Patients TOTAL
	NEG	ASC	LSIL	NC	REG	PRO	CIN3	<CIN3	
6	8	0	0	8	0	0	0	0	8
11	1	0	0	1	0	0	0	0	1
16	42	14	7	34	6	23	8	17	63
18	8	4	2	5	6	3	0	2	14
31	8	0	1	2	1	6	1	2	9
33	2	2	0	1	1	2	0	0	4
35	3	2	4	3	5	1	0	1	9
39	5	2	0	2	2	3	0	2	7
45	10	0	2	6	0	6	3	5	12
51	0	0	0	0	0	0	0	0	0
52	14	3	1	11	0	7	3	4	18
53	1	0	3	2	0	2	0	2	4
54	18	2	2	18	3	1	0	2	22
55	3	2	0	3	1	1	0	0	5
56	0	0	0	0	0	0	0	0	0
58	1	1	3	2	2	1	0	2	5
59	9	1	2	8	2	2	0	1	12
61	6	0	0	5	0	1	0	0	6
62	2	0	1	3	0	0	0	0	3
66	0	0	2	1	0	1	0	1	2
68	0	0	0	0	0	0	0	0	0
70	8	0	0	6	0	2	0	2	8
72	4	0	0	4	0	0	0	0	4
73	2	0	4	4	1	1	0	1	6
81	4	0	0	4	0	0	0	0	4
83	1	0	0	1	0	0	0	0	1
84	2	0	0	2	0	0	0	0	2
Total	162	33	34	136	30	63	15	44	229

Note: NC = no change in class; REG = regress to lower class; PRO = progress to higher class.

In the 229 patients with a persistent HPV infection and a Pap cytology <HSIL, 166 (72.5%) showed no changes or a regression in the follow-up Pap cytology while 63 (27.5%) showed cytologic progression from a lower class to a higher class. Colposcopic biopsies confirmed that the cervical lesion in 15 patients with persistent infection by HPV-16, -31, -45 or -52 progressed to CIN3. These biopsies were performed because the cytology was found to progress to HSIL on a follow-up Pap test or because detection of the same high-risk HPV genotype associated with an unresolved LSIL or ASCUS cytology on repeat tests. Of the 63 patients with a persistent HPV-16 infection, 23 (36.5%) showed Pap cytology progression from a lower class to a higher class (negative > ASCUS > LSIL > HSIL), 6 (9.5%) regressed from a higher class to a lower class (negative < ASCUS < LSIL), and 34 (54%) showed no class changes in the follow-up cytology. The corresponding numbers in these cytologic change categories for the patients with HPV-31 persistent infection were 6 (66.7%), 1 (11.1%) and 2 (22.2%); those with HPV-45 were 6 (50%), 0 (0%) and 6 (50%); and those with HPV-52 were 7 (38.9%), 0 (0%) and 11 (61.1%), respectively. Patients with persistent infections by HPV-18, -35, -39, -53, -54, -58,-59, -59, -66, -70, and -73 did not develop CIN3 or more severe lesions although 16 patients with persistent infections caused by these HPV genotypes had colposcopic biopsies during follow-ups which were proven to be below CIN3. While 12.7% (8/63) of the patients with persistent HPV-16 infection developed a biopsy-proven CIN3 lesion, 16.7% (3/18) of the patients with HPV-52 persistent infection developed a CIN3 lesion during this observation period. HPV-52 has been reported to be a leading cancer-causing genotype among certain populations [91,92], but is consistently underdiagnosed by commercial test kits [93,94,95,96]. In our series, HPV-52 was the second most common HPV genotype detected (Table 1). HSIL Pap cytology was found in one patient, namely 4.5% (1/22), in the samples positive for HPV-45 at the point of entrance (Table 1), and this patient was proven to have a CIN1 lesion on colposcopic biopsy (Table 2). However, when the patients with persistent infection were analyzed, 50% (6/12) of the HPV-45 infected patients showed disease progression, and 25% (3/12) of them developed a CIN3 lesion confirmed by colposcopic biopsies; in comparison 12.7% (8/63) of the HPV-16 persistent infections advanced to CIN3 in the follow-up period (Table 3). This finding suggests that while HPV-16 infection may cause a rapid progression of the precancerous process, infections by other carcinogenic HPV genotypes, such as HPV-45 and HPV-52, may take a longer time to reach their point of no return to malignancy. Such possibility cannot be adequately studied until a sensitive and reliable HPV genotyping test is routinely available for patient management and follow-ups as part of a large-scale field research.

According to the U.S. FDA guidance, a commercial test kit for HPV detection and genotyping may be validated against “*an FDA-approved HPV test that detects the same genotypes*”, or validated by “*PCR followed by sequencing of the amplicon*” [64]. Practically, all commercial HPV test kits are for cervical screening, and have been clinically validated against an “FDA-approved HPV test that detects the same genotypes” [97,98,99,100]. Since a critical feature for high-risk human papillomavirus (hrHPV) test kits for cervical screening is their clinical accuracy for detecting CIN2+ lesions [82,101] and since the Digene HC2 HPV test kit was the first FDA-approved for that purpose [12], all other commercial test kits were subsequently compared in clinical performance with the original Digene HC2 test kit serving as the industrial “gold standard” for evaluation [97,98,99,100]. However, the pathology of CIN2 lesions is an equivocal mixture of morphological features and certainly cannot be used as a standard to validate the results of HPV genotyping based on DNA sequence alignment algorithms of a highly conserved segment of the L1 gene [39]. As a result, very little useful data have been generated by these HPV test kits for further understanding of the effects of persistent infection caused by various HPV genotypes or subtypes which are generally defined as at least one repeat follow-up detection of the same genotype or subtype of HPV six months or longer after the first entrance test [102].

In this series, 372 of the 770 patients who were positive for HPV at entrance (Table 1) were found to be negative for HPV and negative for Pap cytology on repeat tests between 6 months and 3 years after the first sample was tested, indicating a transient nature of the initial HPV infection without carcinogenic consequences. In addition, in 25 of these 770 initially HPV-positive patients the follow-up samples contained an HPV genotype which was different from the initial genotype found at entrance. All of the Pap cytology results on the follow-up samples from these 25 patients with transient HPV infection were negative. The number of these cases was considered too small for further useful analysis. This observation is consistent with the experience of other investigators that HPV genotype shift indicates transient infection [4].

Although HPV persistence is consistently and strongly associated with CIN2-3/HSIL+ lesions, the magnitude of association may vary by duration of persistence, testing interval and HPV genotypes. Precise definition and standardization of HPV testing, sampling procedure, and test interval are needed for reliable clinical testing [101]. There are supporters of using HPV DNA test kits which can predict a spectrum of CIN2/3 histological changes with a 70%–90% accuracy as “proxy” to follow persistent infections [103]. The data presented in Table 3 of this series show that the progression of intraepithelial lesions in persistent HPV infections may be genotype-determined, and such information cannot be provided by a proxy pooled HPV repeat test without accurate genotyping.

## 3. Experimental Section

As stated above, the 8461 alcohol-preserved liquid-based cervicovaginal Pap cytology specimens were collected by board-certified gynecologists in private practice in the town of Milford for HPV testing according to the American College of Obstetricians and Gynecologists guidelines [78].

The HPV tests were ordered as part of regular medical office women’s health care for 6011 patients aged 30 years and older (median age of 44 years, range 30–69 years) as adjunctive screening to routine Pap cytology, and for 2450 patients below 30 years (median age of 24 years, range 16–29 years) as adjunctive screening to Pap cytology or after the patients were found to have a cytologic diagnosis of atypical squamous cells of undetermined significance (ASCUS) or more severe cytological changes. Since most of the patients below 30 were known Pap cytology-positive before the HPV testing as a result of insurance payment policy, the HPV-positive rate in this younger age group was higher than those patients aged 30 and older. This study was a retrospective analysis of laboratory data and the data analysis presented here did not influence the decision of patient management. The lifestyle and genetic makeups of these private patients may be different from those participating in other research projects, and may influence the data presented in this article. As previously reported, Milford has a rural and suburban population of over 50,000, and 93.6% are non-Hispanic white residents, and publication of these laboratory data with blinded patient identities was approved by the Milford Hospital Institutional Review Board as an ongoing research project [66].

The laboratory performing the diagnostic HPV tests on these clinical samples is certified by the State of Connecticut Department of Public Health under the Clinical Laboratory Improvement Amendments of 1988 (CLIA) for human papillomavirus (HPV) by PCR and DNA sequencing based on methods previously published [38,65,66,67]. The routine protocol is summarized as follows.

### 3.1. Initial Sample Processing

After a specimen was received in the laboratory, about 1 mL (5%) of the ThinPrep, or 0.5 mL (5%) of the SurePath cell suspension, was transferred to a 1.5 mL plastic tube for HPV testing. The remaining liquid-based specimen was forwarded to an independent commercial laboratory (Quest Diagnostics Laboratory, Wallingford, CT, USA) for Pap cytology screening. The abnormal cytology was classified and reviewed by a board-certified pathologist as atypical squamous cells of undetermined significance (ASCUS), low grade squamous intraepithelial lesion (LSIL), or high grade squamous intraepithelial lesion (HSIL). The 4-quadrant colposcopic cervical biopsies, if performed, were processed in the department of pathology, Milford Hospital and the histologic sections were interpreted by the hospital pathologist, and the cases were classified as normal, cervical squamous intraepithelial neoplasia 1 (CIN1), cervical squamous intraepithelial neoplasia 2 (CIN2), or cervical squamous intraepithelial neoplasia 3 (CIN3, equivalent to cervical carcinoma *in situ* in the practice of gynecologic pathology). All CIN1, CIN2 and CIN3 lesions are considered to be precancerous, not a life-threatening disease unless progressing further to invasive cancer.

The alcohol-preserved cells in the 1.5 mL plastic tubes were pelleted by a 13,000 rpm (~16,000 × g) centrifugation for 5 min in a Microcentrifuge 5424 w/rotor 022620444 (Eppendorf NorthAmerica, Westbury, NY, USA), and washed first in reagent grade water, then in 1 mL buffer consisting of 50 mM Tris-HCl, 1 mM EDTA, 0.5% Tween 20, pH 8.1. The washed cell pellet was re-suspended and digested at 45–55 °C overnight in 100 μL of 0.1 mg/mL proteinase K (Sigma Chemical Co., St. Louis, MO, USA) dissolved in the same washing buffer. After denaturing the proteins in the cell digestate in a metal block heated to 95 °C for 10 min and a final centrifugation of the digestate at 13,000 rpm for 5 min, the supernatant was used for HPV DNA PCR amplification without further purification.

### 3.2. Primary PCR with MY09 and MY11 Degenerate Primers

For primary PCR amplification, 1 μL of the crude proteinase K digestate, 1 μL of 10 μmolar MY09 primer (5'-CGTCCMARRGGAWACTGATC-3'), 1 μL of 10 μmolar MY11 primer (5'-GCMCAGGGWCATAAYAATGG-3') and 2 μL of water were added to a PCR tube containing 20 μL of LoTemp™ HiFi^®^ DNA polymerase ready-to-use mix (HiFi DNA Tech, LLC, Trumbull, CT, USA) which contained all the components needed for low temperature PCR, including dNTPs, Mg++, buffer, HiFi^®^ DNA polymerases, dsDNA melting agents and dNTP preservatives, to reach a final 25 μL reaction volume. For thermocycling, the temperature steps of a TC-412 Thermal Cycler (Techne Incorporated, Burlington, NJ, USA) were programmed for an initial heating at 85 °C for 10 min, followed by 30 cycles at 85 °C for 30 s, 40 °C for 30 s, and 65 °C for 1 min. The final extension was 65 °C for 10 min.

### 3.3. Heminested PCR with GP6 and MY11 Consensus General Primers

For heminested PCR, a “trace” of the MY09/MY11 PCR products was transferred by a micro-glass rod of about 1.5 mm in diameter with clean wettable surface to a second PCR tube containing 25 μL of complete nested PCR reaction mixture consisting of 20 μL of LoTemp™ HiFi^®^ DNA polymerase ready-to-use mix, 1 μL of 10 μmolar GP6 primer (5'-GAAAAATAAACTGTAAATCA-3'), 1 μL of 10 μmolar MY11 primer and 3 μL of water to generate a ~190 bp nested PCR product for detection by standard gel electrophoresis. The thermocycling steps for heminested PCR were identical to those used for primary PCR.

### 3.4. Direct DNA Sequencing for HPV Genotyping

The positive heminested PCR products were subjected to direct automated DNA sequencing without further purification. Briefly, a trace of the nested PCR products was transferred from the PCR tube with a calibrated micro-glass rod described above into a reaction mixture containing 1 μL of GP6 sequencing primer, 1 μL of BigDye Terminator (v 1.1/Sequencing Standard Kit, Applied Biosystems, Foster City, CA, USA), 3.5 μL 5× buffer, and 14.5 μL of water in a total volume of 20 μL. This reaction mixture was subjected to 20 enzymatic primer extension/termination reaction cycles, according to the protocol supplied by the manufacturer (Applied Biosystems). The final reaction mixture was loaded in an automated ABI 3130 four-capillary genetic analyzer for sequence analysis. A computer-generated base calling electropherogram usually shows an unambiguous sequence of about 70 bases (Figure 1 and Figure 2) for accurate genotyping when the sample was infected with a single HPV genotype. Sequence alignments were performed against the standard HPV L1 gene sequences stored in the GenBank database by BLAST analysis for final validation of the HPV genotyping. An exclusive “100% identities” match between the query and subject sequences, returned by the on-line algorithm, was required for genotyping except for variants not yet recorded in the GenBank.

### 3.5. Negative and Positive Controls

One μL of each digestate sample was placed in a separate PCR tube with a β-globin primer pair for human genomic DNA amplification as a control of specimen adequacy. Specimens with no β-globin gene amplification were excluded as insufficient. All HPV-negative samples had a concomitant positive β-globin gene amplification.

The purified full-length plasmid DNAs of HPV types-6B, -11, -16, and -18 purchased from American Type Culture Collection (ATCC) were used as HPV DNA standards. The heminested PCR products generated of these standard genotypes by the primers used were confirmed by direct automated DNA sequencing to be those of the L1 gene DNA of the expected HPV genotype. This preliminary experiment was to demonstrate that the methodology used in this study was capable of generating genotype-specific template suitable for DNA sequencing.

The purified HPV type-16 DNA was used as the routine positive control and molecular grade pure water instead of DNA extract was used as negative control for each PCR run. To avoid cross contamination, three separate rooms with no air re-circulation were dedicated to the nucleic acid amplification tests. Two of the rooms were each equipped with a 32” PCR workstation (AirClean Systems, Raleigh, NC, USA). All pre-amplification procedures were performed in PCR station I. All post-PCR procedures were carried out in PCR station II, including preparations for the nested PCR and sequencing reaction. Gel electrophoresis and DNA sequencing were performed in the third isolation room. No post-PCR materials or any items potentially contaminated by amplicons or equipment used in the post-PCR rooms were allowed to enter the pre-PCR working space. Transferring of PCR products from one test tube to another to initiate a new chemical reaction was always accomplished by a micro-glass rod to avoid cross contamination due to PCR amplicon aerosol which may occur during micro-pipetting.

## 4. Conclusions

In spite of our newly gained knowledge on the viral etiology in cervical carcinogenesis, a proposed policy depending on prophylactic vaccination of adolescents against carcinogenic HPV infections as a primary means to prevent cervical cancer in developed countries [10] is being challenged [104,105], and has not been considered to be dependable with certainty [26,27]. Industrial attention has focused on exploitation of viral DNA detection technologies as a more sensitive cervical screen for precancerous pathologies in cervical cancer prevention. An “extremely sensitive” HPV DNA test has been recommended to be the screening tool to rule out risks of developing cervical cancer [78,79]. However, after The American Society for Colposcopy and Cervical Pathology (ASCCP), a society whose members perform colposcopies and promote HPV vaccines for cancer prevention [106] entered the virology-based cancer prevention industry, it quickly found out that if a single “extremely sensitive” HPV DNA test were used as the tool for referring women to colposcopic biopsies, the number of excessive and unnecessary 4-quadrant cervical biopsies would be increased to a grossly unacceptable level. As a result, man-made “gold standards” were introduced to adjust the cutoff points of the HPV nucleic acid tests to fit the targets of clinical sensitivity and clinical specificity which are based on a set of ill-defined morphological changes for validation of the nucleic acid assay kits [82]. While there is a DNA sequencing gold standard for reliable HPV genotyping [64,107,108], a technology following the laws of physics, there is no correspondent gold standard in the CIN2, CIN2+, CIN3 and CIN3+ histologic classification system because these morphological changes have no clear demarcations between the individual classes, or as a group [20,21,83,84,85]. Pap smear cytology is widely used and based on evaluation of cell morphology by pathologists, *i.e.*, highly trained medical doctors. It cannot be replaced by a virology test in spite of the ASCCP guidelines which recommended referral to colposcopy of HPV-16/HPV-18+ women with negative cytology [45]. To avoid excessive and unnecessary harmful cervical biopsies, highly sensitive, type-specific or perhaps even variant-specific methods must be used in routine HPV testing for patient management to reliably distinguish persistent HPV infections from transient HPV infections if a viral DNA test is to be implemented as a primary cervical screen tool in conjunction with the traditional Pap smear cytology.
